# Experiment and Numerical Simulation of Damage Progression in Transparent Sandwich Structure under Impact Load

**DOI:** 10.3390/ma15113809

**Published:** 2022-05-27

**Authors:** Mufei Wang, Yuting Li, Haoshun Luo, Xiaoxia Zheng, Zhiqiang Li

**Affiliations:** 1 Institute of Applied Mechanics, Taiyuan University of Technology, Taiyuan 030024, China; wangmufei1030@163.com (M.W.); zhangshu@163.com (Y.L.); robertl@foxmail.com (H.L.); 2 Key Laboratory of Material Strength and Structural Impact, Taiyuan 030024, China; 3 National Demonstration Center for Experimental Teaching of Mechanics, Taiyuan 030024, China; 4 College of Aeronautics and Astronautics, Taiyuan University of Technology, Taiyuan 030024, China; zhengxiaoxia@tyut.edu.cn

**Keywords:** transparent sandwich structure, impact load, crack propagation, impact experiment, numerical simulation, peridynamics

## Abstract

Crack initiation and propagation is a long-standing difficulty in solid mechanics, especially for elastic brittle materials. A new type of transparent sandwich structure, with a magnesium–aluminum spinel ceramic glass as the outer structure, was proposed in this paper. Its dynamic response was studied by high-speed impact experiments and numerical simulations of peridynamics under impact loads, simultaneously. In the experiments, a light gas cannon was used to load the projectile to 180 m/s, and the front impacted the transparent sandwich structure. In the numerical simulations, the discontinuous Galerkin peridynamics method was adopted to investigate the dynamic response of the transparent sandwich structure. We found that both the impact experiments and the numerical simulations could reproduce the crack propagation process of the transparent sandwich structure. The radial cracks and circumferential cracks of the ceramic glass layer and the inorganic glass layer were easy to capture. Compared with the experiments, the numerical simulations could easily observe the damage failure of every layer and the splashing of specific fragments of the transparent sandwich structure. The ceramic glass layer and the inorganic glass layer absorbed the most energy in the impact process, which is an important manifestation of the impact resistance of the transparent sandwich structure.

## 1. Introduction

With the development of aerospace technology, simple transparent parts have struggled to meet their needs in extreme environments. To improve the impact resistance of glass, sandwich panels emerged as a new type of glass structure. Prasad [1] considered double cantilever beam and shear-fracture specimens, employing aluminum facings bonded by two types of adhesives, using the finite element method to analyze its dynamic response. To improve the bonding properties of sandwich structures, Burlayenko [2] carried out free-vibration analyses of sandwich plates with honeycomb and PVC foam cores containing single/multiple debonding. It is of great significance to study the dynamic response of this sandwich structure. Yu [3] established a sandwich diffusion model to predict the moisture absorption behavior of flax/glass-hybrid composites with different stacking sequences. Funari et al. [4] presented a nonlinear approach to investigate the behavior of composite sandwich structures with transversely compressible cores under static and dynamic loading conditions. Moreover, Funari et al. [5] proposed a coupled ALE-cohesive approach to study the dynamic debonding in a layered structure. Glass structures [6] often undergo crack propagation and splashing fragmentation as damage behavior under an impact load. The current research on glass structures includes three parts: theoretical analysis, experiments, and numerical simulations. For theoretical analysis, Krauthammer et al. [7] used the equivalent thickness and equivalent stiffness to simplify a glass structure into an equivalent single-degree-of-freedom mass–spring model and studied the dynamic response of the negative pressure stage under an explosion load. For experiments, Hooper et al. [8] conducted field explosion tests on large-scale laminated glass and pointed out that the dynamic response of laminated glass was divided into five stages. However, in terms of numerical simulations, accurate simulations of crack propagation area problem that has puzzled researchers for a long time. The reason for this is that traditional finite-element methods are based on the continuity of space and the mutual contact of local nodal forces for modeling, which inevitably cause a singularity of the crack tip when dealing with discontinuous problems [9]. It is difficult to simulate the initiation, propagation, bifurcation, and other behaviors of cracks in glass materials under impact load, and additional damage criteria need to be introduced. Peridynamics (PD) [10,11,12] is modeled with the idea of non-local interactions as an emerging theoretical system, which avoids dealing with the discontinuous problems in the continuum mechanics. Wu et al. [13] used conventional state-based PD to simulate the dynamic fracture of laminated glass under a low-velocity load. Hu et al. [14] used experiments and PD simulations to analyze the damage of laminated glass sheets under an impact load, and the results of both studies are consistent.

A new type of multi-layered transparent sandwich structure, which is made from magnesium–aluminum spinel ceramic glass [15]/inorganic glass/plexiglass (PMMA) and bonded by polyurethane (PU), is proposed in this paper. To understand the impact resistance of the transparent sandwich structure, its crack propagation behavior is predicted by experiments and numerical simulations under an impact load. The present paper is organized as follows. In Section 2, a transparent sandwich structure is introduced. In Section 3, a light gas cannon is used for the impact experiments, focusing on observations of the crack propagation mode of the structure. In Section 4, a discontinuous Galerkin PD method is used to study the dynamic responses of crack propagation and failure morphology under an impact load. Finally, the conclusions are summarized in Section 5.

## 2. Transparent Sandwich Structure

**Figure 1:**
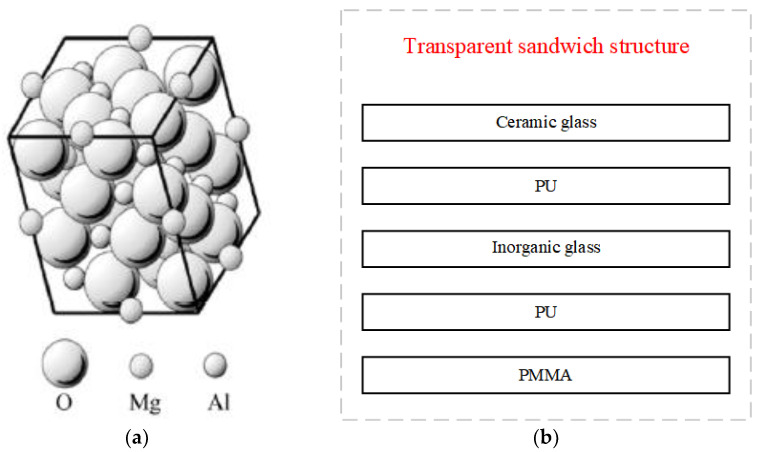
(**a**) Microscopic diagram of magnesium–aluminum spinel; (**b**) A new type of transparent sandwich structure.

Transparent magnesia–aluminum spinel ceramics originated in the 1970s. The latest research on transparent ceramics mainly focuses on the preparation of high-purity magnesia–aluminum spinel powder, the sintering process of transparent ceramics, the influence of additives in the sintering process, and the modification of doping components [16]. The structure of the magnesium–aluminum spinel (MgAl_2_O_4_) belongs to the cubic crystal system. The larger oxygen elements occupy a cubic close-packed lattice, while the smaller magnesium and aluminum elements occupy the voids of the cubic system. This structure ensures the stability of the magnesia–alumina spinel without phase transition, even at high temperatures. The microscopic schematic diagram is shown in Figure 1a.

A new type of transparent sandwich structure is proposed in this paper, as shown in Figure 1b. In this structure, a magnesia–aluminum spinel ceramic is used as an outer-layer material to resist high temperatures and impact loads. PMMA is used as an inner-layer material to play a role in safety protection. Other layers use inorganic glass or PMMA as the structural layer to attenuate the shock wave and reduce damage. PU as the intermediate layer makes the three transparent materials into a whole and dissipates the deformation energy through its bonding effect.

## 3. Experiment

### 3.1. Experimental Setup

**Figure 2:**
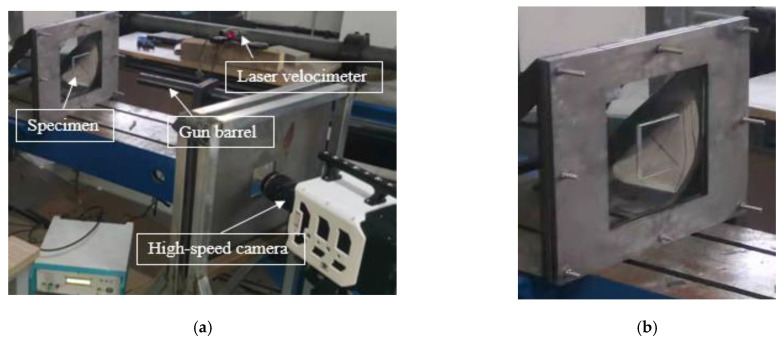
(**a**) Experimental setup; (**b**) Specimen and fixture.

The impact experiments on the transparent sandwich structure were carried out in a laboratory of Taiyuan University of Technology. A light gas cannon was used to accelerate a projectile to a certain speed, and the experimental setup is shown in Figure 2a. The bullet was placed in the straight barrel of the light gas cannon. When the air was compressed to a certain pressure value, the bullet was ejected to impact the target by triggering the device. By controlling the pressure value using the instrument of the device, the projectile could achieve a maximum loading speed of about 300 m/s. After many empty target tests, a relationship between pressure and impact velocity was found. A standard 17.2 g projectile with a 15 mm-long bullet was used in the experiments with an initial velocity of 180 m/s. The steel brackets were fixed by 8 bolt holes, as shown in Figure 2b. To observe the impact process clearly by the high-speed camera, two high-power lamps were placed not far from the specimen for illumination.

### 3.2. Structure Description

**Figure 3:**
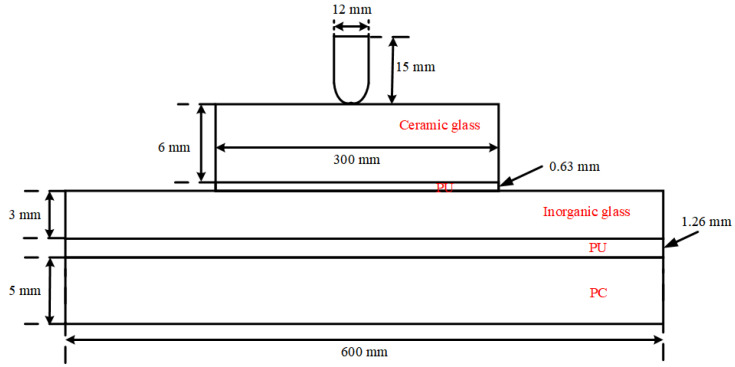
The geometry of the transparent sandwich structure.

The geometry of the transparent sandwich structure and the bullet used in the experiments are shown in Figure 3 (for the convenience of dimensioning, it is not drawn in relative scale). The specimen comprised of five layers of 300 mm × 300 mm magnesium–aluminum spinel ceramic glass of 6 mm thickness, 600 mm × 600 mm inorganic glass of 3 mm thickness, 600 mm × 600 mm PC of 5 mm thickness, and two layers of PU. This structure was separated by 0.63 mm and 1.26 mm of polyurethane, while the lateral edges of the structure were confined with tape. The whole structure was clamped by steel plate.

### 3.3. Experimental Results

The bullet stopped in the ceramic glass layer and was ejected from the target. Due to the hardness of the transparent sandwich structure, the bullet itself was recessed inward by 3 mm. The bullet impact generated strong stress waves that propagate in the transparent sandwich structure. We focused on the damage evolution of the strike face and analyzed the overall damage pattern of the ceramic glass layer and the inorganic glass layer.

#### 3.3.1. Damage Evolution of the Strike Face

**Figure 4:**
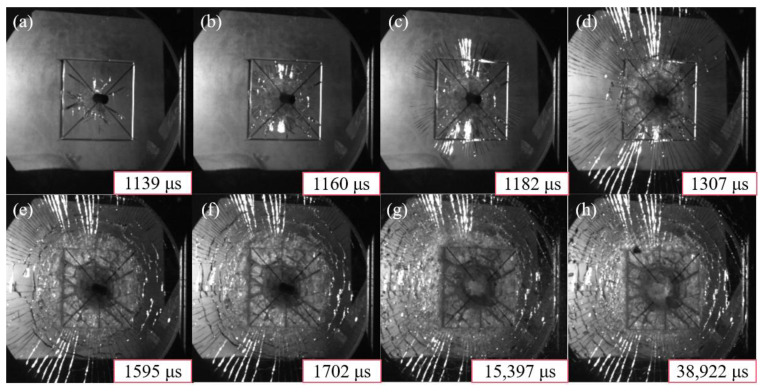
The time evolution of the damage map for the strike face. From (**a**–**h**): snapshots taken at 1139, 1160, 1182, 1307, 1595, 1702, 15,397, and 38,922 μs, starting from the triggering of the high-speed camera.

It was interesting to observe the evolution of damage in the strike face. The high-speed camera effectively captured the whole process of the damage evolution, as shown in Figure 4. We observed that the damage only evolved in the top face of the strike face in the experiment, not the bottom face. Starting from the triggering of the high-speed camera, it can be seen that the bullet contacted the ceramic glass layer at 1139 μs, and the cracks propagated to the boundary of the ceramic glass within a few tens of microseconds. The radial cracks started in the inorganic glass, and they propagated towards the boundaries of this layer until 1307 μs. After more than two hundred microseconds, circumferential cracks began to appear and continued to propagate outwards. Finally, the transparent sandwich structure formed an impact crater with an area of 56.25 cm^2^, and the whole structure was dominated by radial cracks and circumferential cracks.

#### 3.3.2. Ceramic Glass Layer

**Figure 5:**
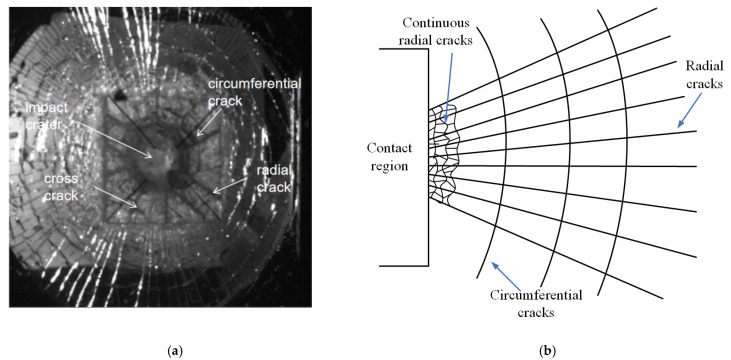
(**a**) Details of damage in ceramic glass layer; (**b**) Schematic drawing of features seen in inorganic glass layer.

The ceramic glass layer was the outermost material that resisted the high-speed projectile; the details of damage are shown in Figure 5a. There was a visible crater at the impact center. However, there was no large area falling off due to the bonding effect of the PU layers, and there were dozens of fine glass fragments only around the specimen. Several circumferential cracks appeared in the annular area (55–80 mm) away from the center of the impact crater. In the annular area (80–150 mm) away from the center of the impact crater, a total of 11 relatively radial cracks appeared, and the widths of the radial cracks were significantly larger than that of the inorganic glass layer. In addition, several cross-type cracks appeared at the endpoints of radial cracks. In general, the ceramic glass layer had smashing damage at the crater after absorbing a large amount of kinetic energy from the bullet. The number and types of cracks in the ceramic glass layer were lower when compared with the inorganic glass layer, which reflected the excellent impact resistance of the ceramic glass layer.

#### 3.3.3. Inorganic Glass Layer

The inorganic glass layer acts as a structural layer to attenuate the shock wave, reducing the damage to the protection target and further absorbing the impact energy brought by the projectile. Figure 5b shows the failure form of the inorganic glass layer in a schematic drawing. Compared to the failure mode of the ceramic glass layer, one of the most remarkable features of the inorganic glass layer was the increase in the number of circumferential cracks and radial cracks. Due to the inorganic glass layer and the ceramic glass layer being bonded by the PU material, the failure form of the joint could not be directly observed. A large number of fine continuous radial cracks could be seen where the ceramic glass layer extended outward by 30 mm. In addition, the surface cracks on the inorganic glass layer propagated in a local fan shape, no complete circumferential cracks appeared, and the continuity of the cracks was poor.

## 4. Numerical Simulation of Peridynamics

### 4.1. Model Description

**Figure 6:**
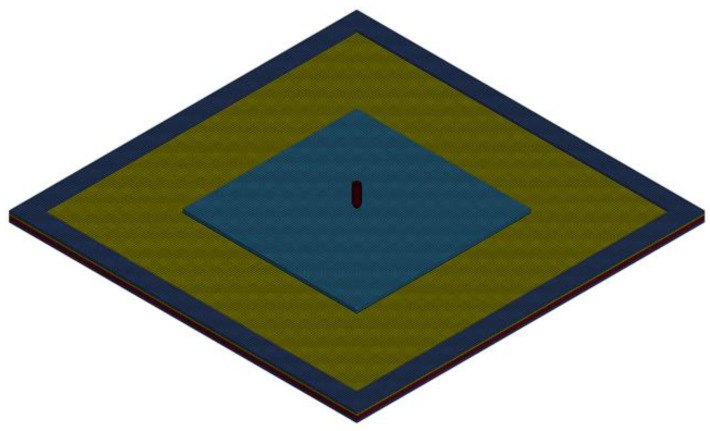
Computational model of transparent sandwich structure.

The transparent sandwich structure and the projectile were parametrically modeled by TrueGrid software (Version 3.1.3, XYZ Scientific Applications, Pleasant Hill, CA, USA), and the geometry of the entire calculation model is the same as that in Figure 3. The in-plane mesh size of the transparent sandwich structure is 3 mm. The ceramic glass layer, inorganic glass layer, and PC layer are divided into two meshes in the thickness direction to consider the bending effect. The computational model of the transparent sandwich structure is shown in Figure 6. To use the PD method in the LS-DYNA software, it is necessary to separate the nodes of each glass element and its adjacent glass elements, so that they cannot share the nodes of the element similar to those used in classical continuous mechanics. According to the trial calculation results, it is necessary to ensure that the element nodes at the frame and the adhesive layer coincide with the glass element nodes. Otherwise, there will be a large number of particles splashing at the non-overlapping places, and the true failure mode of the material cannot be judged. In addition, to ensure that the ceramic glass layer cannot deflect during the impact process, the degrees of freedom at the boundary of the ceramic glass layer need to be completely constrained.

### 4.2. Material Model

In this paper, the discontinuous Galerkin bond-based peridynamics (DG-PD) method [17] was used to simulate the impact resistance of the transparent sandwich structure, which required the node separation operation of the ceramic glass layer, inorganic glass layer, and PC layer. The node separation operation increases the number of nodes, and the calculation costs significantly increase compared with the element deletion method. In the constitutive model of bond-based PD, the threshold value of the critical strain energy release rate *G*_t_ needs to be set, and *G_t_* is directly related to the critical elongation rate. When the elongation exceeds the critical value, the bonds between the point pairs no longer interact and cannot be recovered [18]. The model parameters of the ceramic glass layer, inorganic glass layer, and PC layer material are shown in Table 1.

In Table 1, *ρ* represents the mass density; *E* represents the elastic modulus; *G*_t_ represents the critical strain energy release rate; and *HSFAC* represents the normalized support domain size. LS_DYNA automatically adjusts the value of *HSFAC* to ensure a suitable horizon of the material point. Here, the most important parameter is the selection of the critical strain energy release rate.

The PU layer is a common polymer with an excellent viscosity and large deformation properties that can be studied as a viscoelastic plastic or hyper-elastic material in numerical simulations. In the laminated glass structure, the PU layer can effectively make up for the shortcomings of the small deformation of the glass layer. It plays an important buffering role in the impact problem and can reduce the splashing of glass fragments to a certain extent. In this paper, the PU layer adopts a hyper-elastic material model, and the mechanical parameters of the model are shown in Table 2.

In Table 2, *ρ* represents the mass density; *μ* represents the Poisson’s ratio; and *A* and *B* represent the Rivlin constant of the adhesive layer material, which can be determined by a uniaxial tensile test.

Both the frame and the bullet are made of steel material, which is simplified as the rigid body material model in this paper. Table 3 shows the parameters of the frame and the bullet material model.

### 4.3. Simulation Results and Analysis

The discontinuous Galerkin PD method is used to simulate the dynamic response of the transparent sandwich structure, focusing on the analysis of the crack propagation and failure behavior of the ceramic glass layer and inorganic glass layer. In contrast with the experiments, the simulation results can allow us to independently observe the damage evolution of each layer of the transparent sandwich structure. This important advantage of numerical simulation helps us to study the impact resistance of multi-layered brittle systems under extreme loading conditions.

#### 4.3.1. Damage Evolution in the Ceramic Glass Layer

**Figure 7:**
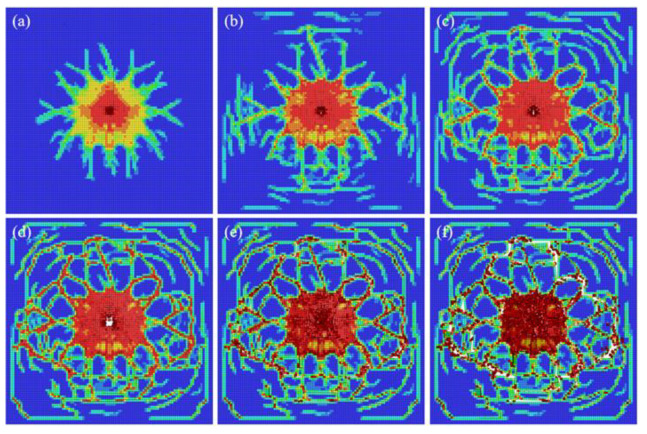
The time evolution of the damage map for the ceramic glass layer. From (**a**–**f**): snapshots taken at 40, 60, 80, 220, 840, and 2000 μs from the contact between the bullet and the transparent sandwich structure.

Figure 7 shows the damage evolution process of the ceramic glass layer under a high-speed impact of 180 m/s. It can be seen that the final simulation results and the experimental results in Figure 5a have a good fit. Not only is the crater morphology at the impact center presented but multiple radial cracks are also captured in the experiment. However, due to the different setting of boundary conditions, the length and number of cracks are slightly different between experiments and simulations. From the point of view of the damage evolution process, cracks are generated within tens of microseconds, continue to expand outwards, and form more cracks in the back-and-forth reflection of waves after a few milliseconds. From the perspective of the damage form, the radial cracks and circumferential cracks intersect and penetrate each other in the ceramic glass layer, which effectively captures the behavior of multi-crack interaction.

#### 4.3.2. Damage Evolution in the Inorganic Glass Layer

**Figure 8:**
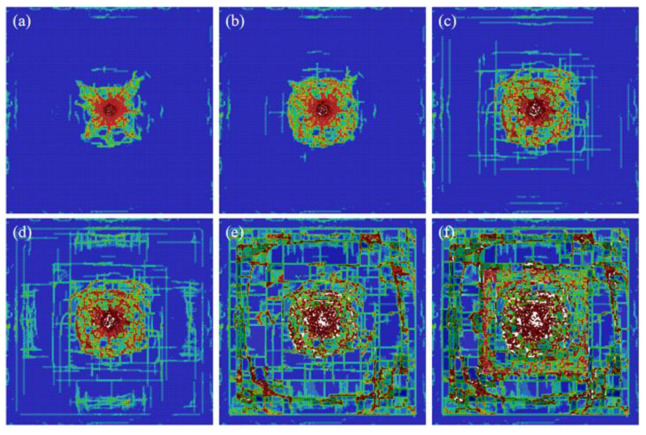
The time evolution of the damage map for the inorganic glass layer. From (**a**–**f**): snapshots taken at 40, 60, 80, 220, 840, and 2000 μs from the contact between the bullet and the transparent sandwich structure.

Figure 8 shows the damage evolution process of the inorganic glass layer under a high-speed impact of 180 m/s. The damage of the inorganic glass layer has a hysteresis compared to that of the ceramic glass layer at the same time. There is no obvious radial crack, but the damage mode at the contact between the inorganic glass layer and ceramic glass layer is captured. When the bullet passes through the ceramic glass layer and the PU layer, the kinetic energy is significantly reduced, causing the damage to the inorganic glass layer to be lower than that of the ceramic glass layer. Due to the contact and boundary conditions between the inorganic glass layer and ceramic glass layer, another obvious feature is that the final damage mode of the inorganic glass layer has the approximate shape of the ceramic glass layer.

#### 4.3.3. Splashing of Glass Fragments

**Figure 9:**
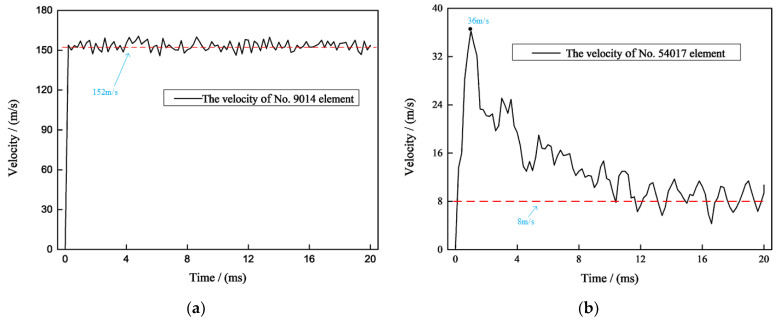
(**a**) No. 9014 ceramic glass element speed change diagram; (**b**) No. 54017 inorganic glass element speed change diagram.

The transparent sandwich structure will produce a large number of glass fragments under the high-speed impact of the projectile, which may cause certain damage to nearby people. Therefore, it is necessary to select some typical glass elements to study their splashing velocity. Figure 9a shows the speed change diagram of the No. 9014 element, which is the center element of the ceramic glass layer. This element increases to 152 m/s in an extreme time under the impact of the projectile and then maintains a slight oscillation within tens of microseconds.

Figure 9b shows the speed change diagram of the No. 54017 element, which is the center element of the inorganic glass layer. This element increased to a maximum speed of 36 m/s at 1.5 ms and then oscillated back to around 8 m/s. Due to the inorganic glass layer being located in the middle of the transparent sandwich structure, the glass fragments were affected by the glue layer and other glass layers during the splashing process, resulting in a reduction in their kinetic energy. In addition, from the maximum velocities of the ceramic glass layer and the inorganic glass layer, the energy absorbed by the ceramic glass layer clearly constitutes the main part.

#### 4.3.4. Energy Absorption

**Figure 10:**
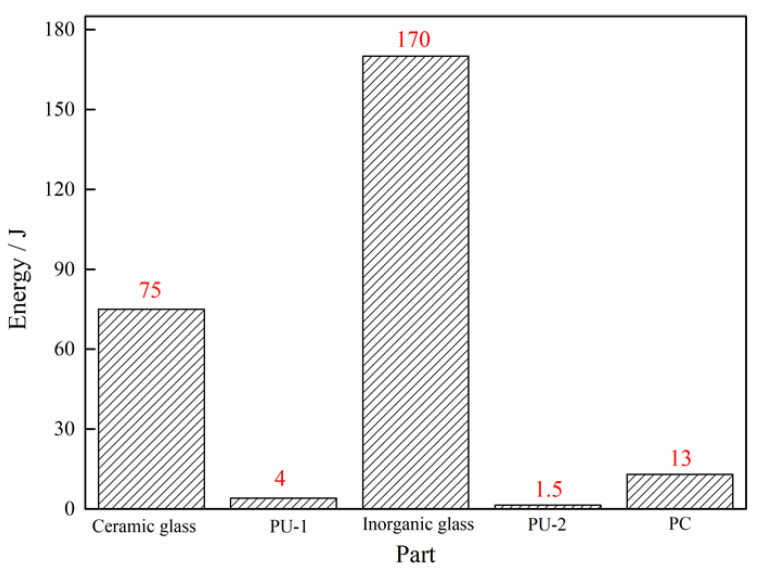
The mechanical energy of each part at 20 ms after the projectile hits.

During the high-speed impact of the projectile on the transparent sandwich structure, the kinetic energy of the projectile is converted into the kinetic energy and internal energy of each component of the transparent sandwich structure, which is the internal cause of structural damage. Taking the sum of the kinetic energy and internal energy of the components as the total energy, Figure 10 shows the total energy histogram of each component at 20 ms after the impact. It can be seen that the inorganic glass layer and ceramic glass layer absorb most of the energy from the projectile. However, the area of the ceramic glass layer is only 1/9 of the inorganic glass layer. Therefore, the relative energy absorption accounts for the largest proportion in the ceramic glass layer. The PU-1 and PU-2 adhesive layer absorb only 1.52% and 0.57% of the energy, respectively, but they effectively prevent the splashing of glass fragments. In addition, the proportion of energy absorbed by the PC layer is 4.93%. In summary, the ceramic glass layer and the inorganic glass layer are the main components of the transparent sandwich structure to resist the impact of the projectile. Together with the bonding effect of the PU adhesive layer and the supporting effect of the PC layer on the backplane, the excellent impact resistance of the transparent sandwich structure is comprehensively exerted.

## 5. Conclusions

A new type of transparent sandwich structure with magnesium–aluminum spinel ceramic glass as the outer structure is proposed in this paper. High-speed impact experiments were carried out by using a light gas cannon test device, and the corresponding numerical simulations of the transparent sandwich structure were adopted by using the discontinuous Galerkin peridynamic method.

The experiments and peridynamic simulations show several main features of crack propagation regarding the impact problems of the transparent sandwich structure: (a) Both the impact experiments and the peridynamic simulations can effectively reproduce the crack propagation process of the transparent sandwich structure. (b) The radial cracks and circumferential cracks of the ceramic glass layer and the inorganic glass layer are obvious. Apart from the crushing damage at the impact crater, the integrity of the rest of the parts is relatively good. (c) Compared with the experiments, the numerical simulations can easily observe the damage to each layer and the splashing of specific fragments. (d) The ceramic glass layer and the inorganic glass layer absorb most of the energy in the impact process, which is an important manifestation of the impact resistance of the transparent sandwich structure.

## Figures and Tables

**Table 1:** The DG-PD material model parameters of the ceramics glass, inorganic glass, and PC.

Material	ρ (kg/m^3^)	E (GPa)	G_t_ (J/m^2^)	HSFAC
Ceramic glass	3580	190	40	0.8
Inorganic glass	2530	72	15.47	0.8
PC	1200	2	4	0.8

**Table 2:** Mechanical parameters of the PU adhesive layer.

ρ (kg/m^3^)	*μ*	A (MPa)	B (MPa)
1100	0.495	1.6	0.06

**Table 3:** The rigid body material model parameters of the frame and the bullet.

ρ (kg/m^3^)	*μ*	E (GPa)
7850	0.28	200

## Data Availability

Not applicable.

## References

[1] Prasad S., Carlsson L.A. (1994). Debonding and crack kinking in foam core sandwich beams—II. Experimental investigation. Eng. Fract. Mech..

[2] Burlayenko V.N., Sadowski T. (2011). Dynamic behaviour of sandwich plates containing single/multiple debonding. Comput. Mater. Sci..

[3] Yu H., Zhou C. (2017). Sandwich diffusion model for moisture absorption of flax/glass fiber reinforced hybrid composite. Compos. Struct..

[4] Funari M.F., Greco F., Lonetti P. (2018). Sandwich panels under interfacial debonding mechanisms. Compos. Struct..

[5] Funari M.F., Greco F., Lonetti P. (2017). Dynamic debonding in layered structures: A coupled ALE-cohesive approach. Frat. ed Integrità Strutt..

[6] Bless S., Chen T. (2010). Impact damage in layered glass. Int. J. Fract..

[7] Krauthammer T., Altenberg A. (2000). Negative Phase Blast Effects on Glass Panels. Int. J. Impact Eng..

[8] Hooper P., Sukhram R., Blackman B., Dear J. (2012). On the Blast Resistance of Laminated Glass. Int. J. Solids Struct..

[9] Banks-Sills L. (2010). Update: Application of the Finite Element Method to Linear Elastic Fracture Mechanics. Appl. Mech. Rev..

[10] Silling S.A. (2000). Reformulation of elasticity theory for discontinuities and long-range forces. J. Mech. Phys. Solids.

[11] Silling S.A., Askari E. (2005). A meshfree method based on peridynamic model of solid mechanics. Comput. Struct..

[12] Silling S.A., Epton M., Weckner O., Xu J., Askari E. (2007). Peridynamic states and constitutive modeling. J. Elast..

[13] Wu L., Wang L., Huang D., Xu Y. (2020). An ordinary state-based peridynamic modeling for dynamic fracture of laminated glass under low-velocity impact. Compos. Struct..

[14] Hu W., Wang Y., Yu J., Yen C.F., Bobaru F. (2013). Impact damage on a thin glass plate with a thin polycarbonate backing. Int. J. Impact Eng..

[15] Benitez T., Gómez S.Y., De Oliveira A.P.N., Travitzky N., Hotza D. (2017). Transparent ceramic and glass-ceramic materials for armor applications. Ceram. Int..

[16] Ganesh I. (2013). A review on magnesium aluminate (MgAl_2_O_4_) spinel: Synthesis, processing and applications. Int. Mater. Rev..

[17] Ren B., Wu C., Askari E. (2017). A 3D discontinuous Galerkin finite element method with the bond-based peridynamics model for dynamic brittle failure analysis. Int. J. Impact Eng..

[18] Bobaru F., Ha Y., Hu W. (2012). Damage progression from impact in layered glass modeled with peridynamics. Cent. Eur. J. Eng..

